# Diagnoses Based on C-Reactive Protein Point-of-Care Tests

**DOI:** 10.3390/bios12050344

**Published:** 2022-05-17

**Authors:** Miroslav Pohanka

**Affiliations:** Faculty of Military Health Sciences, University of Defense, Trebesska 1575, CZ-50001 Hradec Kralove, Czech Republic; miroslav.pohanka@gmail.com or miroslav.pohanka@unob.cz

**Keywords:** immunity, immunoassay, infection, inflammation, lateral flow test, point-of-care test, review

## Abstract

C-reactive protein (CRP) is an important part of the immune system’s reaction to various pathological impulses such as bacterial infections, systemic inflammation, and internal organ failures. An increased CRP level serves to diagnose the mentioned pathological states. Both standard laboratory methods and simple point-of-care devices such as lateral flow tests and immunoturbidimetric assays serve for the instrumental diagnoses based on CRP. The current method for CRP has many flaws and limitations in its use. Biosensor and bioassay analytical devices are presently researched by many teams to provide more sensitive and better-suited tools for point-of-care tests of CRP in biological samples when compared to the standard methods. This review article is focused on mapping the diagnostical relevance of CRP, the applicability of the current analytical methods, and the recent innovations in the measurement of CRP level.

## 1. Introduction

Point-of-care diagnostic tests for measuring molecular markers have become a substantial tool in modern healthcare. The tests are relevant for the early diagnosis of various pathological states and diseases. Various analytical devices have been developed in the past decades founded on the electrochemical and optical (colorimetrical) principle of signal determination. Electrochemical glucose biosensors, chemosensors and nanoparticle-based assays [1,2,3,4], coronavirus disease antigen tests and bioassays [5,6,7], pregnancy tests for human chorionic gonadotropin [8,9,10], and alcohol breath analyzers—breath alcohol testers [11,12] can be introduced as typical examples. There is an effort to gradually grow the number of markers measured this way to create an alternative to the advanced instrumental analyses typical for standard laboratories.

C-reactive protein (CRP) is a standard immunochemical marker being measured to diagnose some types of bacterial and fungal infections, recognize inflammatory and some autoimmune disorders, and cardiovascular diseases [13,14]. The CRP used as a marker has a substantial advantage in high concentrations in the blood of diseased subjects equal to or higher than 10 mg/L. Thus, concentration is easily measurable by simple sensors, handheld assays, and other point-of-care tests. Diagnoses founded on the CRP assay are highly accessible and hardly replaceable by other markers [15].

In recent years, the use of CRP as a marker has become highly popular and many tests have been developed and are also commercially available. The new tests and methods change the old approaches where CRP was taken for a marker measurable by an advanced laboratory technique. This review summarizes point-of-care tests and their applicability to CRP for the routine revealing of pathological states. The diagnostic relevance of CRP is discussed as well. The recent discoveries in this field are written and cited here. This review describes new analytical methods such as various biosensors and bioassays and their applicability for diagnoses by CRP level determination is summarized. This paper also plays a role as an overview of actual literature on CRP diagnostic methods and point-of-care tests because significant progress in the available methods for CRP has been made in recent years.

## 2. The Use of CRP in Diagnoses

**Figure 1:**
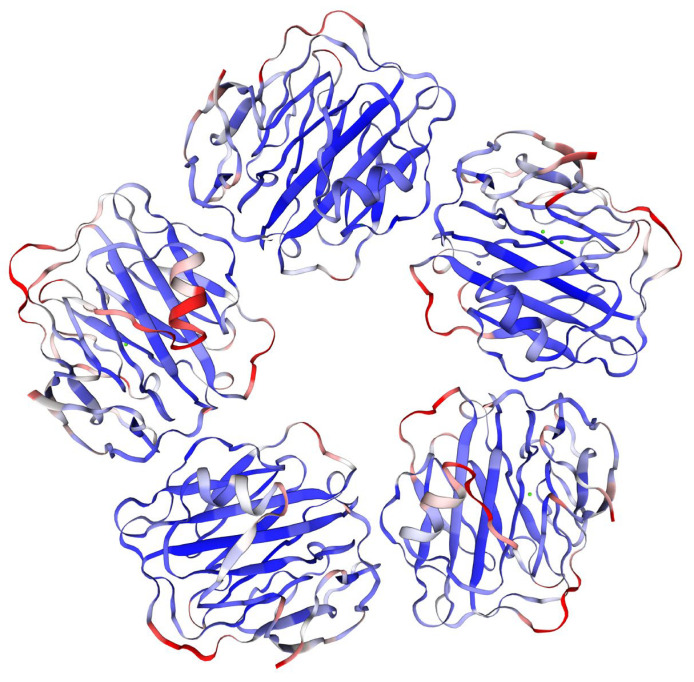
X-ray crystallography structure of a pentameric CRP made by Guillon and coworkers [23] and visualized using SWISS-MODEL online application [24,25,26].

CRP belongs to an evolutionarily conserved protein family of pentraxin with specifications close to the other family members. In humans, the whole molecule weighs around 120 kDa and it comprises five identical subunits (homopentamer), each sized 206 amino acids [16]. The weight of CRP can differ depending on its origin and cited study. Considering the significance of CRP in both human and veterinary medicine, molecular weights of CRP from various organisms should be taken into consideration when a new analytical method for CRP is established with a principle based on molecule or its fragments weighting (e.g., mass spectrometry). The molecular weight of a subunit between 20 and 30 kDa and a total range of 100–150 kDa for the whole molecule can be found in the literature for CRP over various mammals [16,17,18]. The final molecule of CRP is composed of five subunits arranged into a circle plain shape with central space [19,20,21,22]. The pentameric structure of CRP can be determined from X-ray crystallography structures. An X-ray crystallographic structure made by Guillon and coworkers [23] and visualized using the SWISS-MODEL online application [24,25,26] is given in Figure 1.

In the human organism, hepatocytes produce CRP, but other cells such as macrophages, lymphocytes, adipocytes, smooth muscle cells, and epithelial cells can create and release CRP into blood [27]. The production of CRP by hepatocytes previously activated by hepatocyte nuclear factors is the major source of CRP in an organism [28,29].

**Figure 2:**
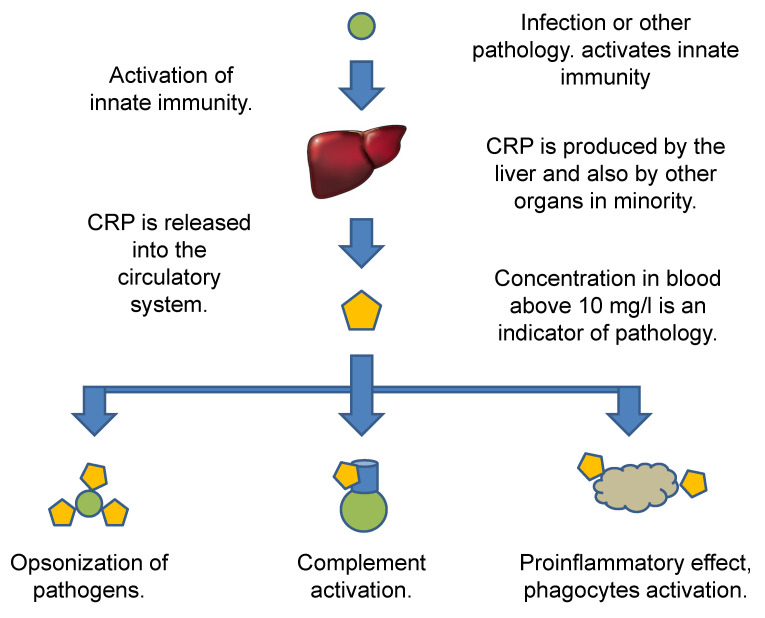
Survey of CRP pathway and initiated effects.

The opsonization of pathogens and target cells is the major task for CRP and it results in the activation of further steps leading to killing pathogens and inactivating damaged cells [30]. The CRP is part of the inflammatory reactions in the organism further stimulating immunity, including macrophages and promoting phagocytosis by professional phagocytes, preventing infections and other malignancies, but the CRP can also be involved in autoimmune disorders [31]. The blood circulating form of CRP is dominantly a homopentamer, which can associate with phosphocholine-rich membranes of immune cells and cells undergoing apoptosis, can dissociate to the subunits that are further involved in both classic and alternative complements, and can activate leukocytes [32]. A survey of the CRP pathway and initiated effects is given in Figure 2.

Monomeric CRP can occur, but the single subunit has a different conformation than the pentameric structure. It cannot firmly bind to the phosphocholine-rich membranes and initiate the proinflammatory pathways [33]. The proinflammatory effect, leukocyte movement, and other characteristics are also different for the monomeric CRP than for the normal pentameric form [34].

The increased level of CRP was proven to be associated with a wide number of diseases and disorders. Heart failures and cardiovascular diseases [35,36], osteoarthritis [37], visceral adiposity [38], poorer memory in elderly women [39], alcohol intake and some dementias [40], non-alcoholic fatty liver disease [41], periodontitis [42,43], appendicitis [44], chronic and systemic inflammation [40,45,46], sepsis [47,48], bacterial infection [49,50,51], some viral infections such as severe coronavirus disease 2019 [52,53], candidiasis [54,55], and some types of cancer [56,57,58] can be associated with the growing level of CRP. Because CRP is more significantly increased due to bacterial infections than the viral ones, CRP tests serve for the decision of antibiotics prescription [59]. The use of CRP as a biomarker for antibiotics indications is dominantly related to respiratory tract infections and it is a nonspecific diagnosis that should be accompanied by a differential diagnosis based on the visual manifestation of an acute infection. On the other hand, the efficacy of bacterial infection diagnosis based on CRP followed by antibiotics prescription is positively accepted by physicians, and the reliability has been verified in several studies [60,61,62,63].

In recent years, it has been recognized that the applicability of CRP for diagnosis can be further enhanced by distinguishing between standard pentameric CRP and monomeric CRP. The monomeric CRP is at an increased level presented in some pathologies, it was recognized in aggregated platelets, and an association with atherosclerosis and atherothrombosis was proposed [32]. Some studies have pointed out that monomeric CRP can be about a growing tumor [64,65]. The monomeric CRP is also a sensitive marker of inflammation [66,67].

Generally, CRP is considered a pathological marker where the intensity of the pathology is proportional to the CRP level in the blood, and concentrations under 10 mg/L are taken for normal and levels above 10 mg/L are indicators of pathology [68]. The threshold level of 10 mg/L nevertheless historically comes from the sensitivity of obsolete immunochemical and point-of-care tests that are not sensitive enough to prove lower concentrations and CRP correlated with pathologies even at levels under 10 mg/L. There are further threshold levels distinguished with values depending on the type of pathology. For instance, Socha et al. described a correlation between CRP above 3.33 mg/L and endometrial cancer [69]. In his study, Ridker proposed thresholds for the risk of cardiovascular diseases as follows: under 1 mg/L of blood CRP is a low risk, 1–3 mg/L is a moderate risk, and a high risk starts at CRP blood levels above 3 mg/L [70]. The distinguishing of CRP levels under 10 mg/L is connected with the introduction of high-sensitivity CRP tests suitable for low concentrations of CRP. These tests are also known under the common abbreviation hsCRP. The aforementioned facts about CRP are summarized in Table 1.

## 3. Routine CRP Point-of-Care Tests and Immunochemical Tests

Standard biochemical and immunochemical methods are known for the determination of CRP levels in the blood. Apart from the highly instrumental methods such as chromatography or mass spectrometry, some simple tests are available for the CRP assay. Simple kits and tests routinely available for clinical use are discussed in this chapter. The enzyme-linked immunosorbent assay (ELISA) can be exampled as a routine method used under laboratory conditions for the CRP assay [71,72,73,74]. Standard kits for ELISA represent a simple way to measure CRP. The ELISA kits can be based on various configurations but the most common type found on 96-well microplates and secondary antibodies labeled with horseradish peroxidase exert a standard applicability range (range of a calibration curve) with a bottom limit of around 25 ng/L (range of the bottom limit between approximately 15 and 50 ng/L of CRP) and an upper limit approximately around 25 µg/L (range of the upper limit from 1 µg/L up to 50 µg/L). The limit of detection of such kits can reach approximately 5 ng/L. The sensitivity of the current kits is between 2 and 40 ng/L but tests with sensitivities above 100 ng/L exist as well and are applicable. The time of the assay depends on the fact of whether the wells are pretreated or the surface has to be coated with antibodies and blocking reagents or the wells should be treated in the laboratory. The pretreated kits can be finished between 90 min and 4 h, while full modification of the wells with the following assay can take 24 h. The precision of such kits expressed by the relative standard error is between 1.5% (8 and more repeats) and around 5% (for 3 repeats), but tests with an inter-assay relative standard error of 12% are sold as well. Cited papers were, for instance, using kits falling into the mentioned specifications [75,76,77]. Though ELISA is sold in the form of simple kits, it is not suitable for use under point-of-care conditions, and qualified staff and laboratory equipment are necessary for the method realization. Hazardous reagents (hydrogen peroxide and sulfuric acid as stop reagents) can occur in the kits. On the other hand, some ELISA readers and kits are quite inexpensive, so they can be placed and operated in small laboratories such as field hospitals, local laboratories, and temporary healthcare laboratories established for a specific purpose such as care in sites of disaster or refugee camps. There also exist other methods with similar specifications, pros, and cons such as ELISA. The chemiluminescent immunoassay (CLIA) can be exampled. It is a common method that is suitable for the analysis of various analytes including CRP [78,79,80]. The method has many similarities with ELISA including the use of enzymes such as peroxidase for antibodies labeling and expected typical users. However, the enzyme converts a substrate into the chemiluminescent compound in the CLIA method instead of colored, which is typical for ELISA. Increased sensitivity can be expected for CLIA when compared to ELISA. A sensitivity around 5 ng/L, test range approximately between 10 ng/L and 10 µg/L, inter-assay relative standard error of 12%, and assay time around 4 h are common specifications for currently available CLIA kits for CRP. Less sensitive CLIA tests with a detection range between 300 ng/L and 20 µg/L and a sensitivity of 190 ng/L are also on the market.

**Figure 3:**
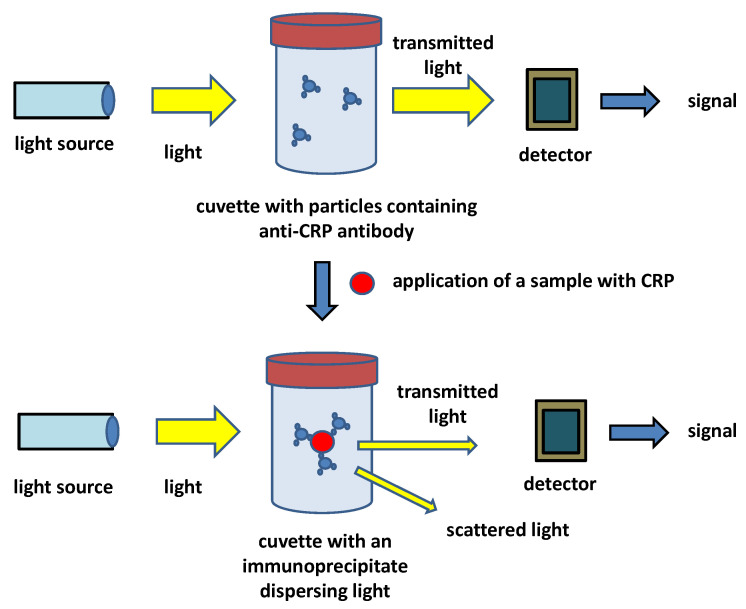
Principle of an immunoturbidimetric assay of CRP.

In addition to the laboratory methods such as ELISA, simple methods to be performed outside laboratories were also searched and simple CRP assays based on immunochemical tests can even be performed in conditions of a small healthcare institution or even in the point-of-care environment. Immunoturbidimetry and immunonephelometry can be exampled as routine methods for those commercial kits are available and many of the kits can be labeled as hsCRP tests due to their analytical specifications [81,82]. While immunonephelometry can be considered a laboratory method rather than a point-of-care one, immunoturbidimetry can be used under laboratory conditions but is also quite common in physician offices and point-of-care conditions. The first CRP kits based on immunoturbidimetry have been developed since the early 1990s. Pioneering work for the determination of CRP in dogs [83], monkeys [84], and humans [85] by immunoturbidimetry can be mentioned. Currently, immunoturbidimetry represents a routine method in human and veterinary medicine for measuring CRP in serum or plasma [86,87,88,89,90,91,92]. Immunoturbidimetry is a simple and low-cost method where direct interaction between an antigen and an antibody is measured by changing solution turbidity. The kits currently used for CRP have either polyvalent antibodies allowing immunoprecipitation or antibodies bound on nano- or microparticles that are frequently prepared from latex, but other materials are used as well. The common principle of an immunoturbidimetric assay of CRP is depicted in Figure 3. The immunoturbidimetric and immunonephelometric assays can be performed on automated laboratory analyzers but there are also available simple and cheap devices suitable for point-of-care conditions. Turbidimetry is, in principle, easier than nephelometry because it measures light intensity in its original axis, and less sensitive and cheaper sensors and less powerful sources of light are necessary for such devices; nevertheless, the turbidimetric devices can in most cases fully compete with the nephelometric one and provide comparable results [93,94,95]. It is the reason why CRP as well as the other immunochemical markers are analyzed by a turbidimetric device rather than the nephelometric one. The immunoturbidimetric methods have worse specifications than the immunoanalytical methods such as ELISA or CLIA but are fully applicable and can be a part of hsCRP tests. Limits of detection for the immunoturbidimetric assay of CRP by commercial tests are in the range of milligrams per liter, typically 2 mg/L, and a calibration range up to 150 mg/L. The time of assay is the major advantage of the tests when compared to ELISA or CLIA as laboratory competitors: the assay can typically be finished within 15 min but some producers claim times under 5 min.

Despite immunoturbidimetric assays being quite simple and cheap to be performed, they still need an analytical device that excludes them from solitary use. Manipulation with an analytical device can be a source of errors in the analytical procedure even if the device is quite simple. Manufacturing simple disposable paper colorimetric devices is the further simplification of the analytical methods to make them more convenient for the establishment of point-of-care tests. Lateral flow tests represent a group of analytical devices being commercially available for many analytes including the CRP. The lateral flow tests are simple colorimetric disposable analytical devices historically coming out from thin-layer chromatography and the first introduction into the market as pregnancy tests in the 1980s followed by the introduction of other analytes including biochemical and immunochemical markers, microorganisms and viruses, various toxins, and other substances [96,97,98,99,100]. The color lines formed in the lateral flow tests can be scaled by the naked eye; therefore, no specific analytical device is needed, resulting in a substantially low price of the assay. The fact that the tests are based on a simple paper or plastic foil cut in combination with a small number of reagents such as antibodies, aptamers, color, luminescence, fluorescence, or nanoparticles labels on the surface is another reason why the lateral flow tests are economically available analytical devices with practical application in various studies [101,102,103,104]. The lateral flow tests have disadvantages as well. When compared to the aforementioned methods, the lateral flow tests are highly dependent on the subjective scaling of coloration. The colored lines are either formed or not visible at all. Some tests can be further improved by scaling color line intensity, but the lateral flow tests are, in most cases, qualitative only. Full quantification by lateral flow tests is not common and semiquantitative assays based on a colorimeter and a lateral flow test are probably the upper improvement of the qualitative lateral flow tests using the naked eye only. The lateral flow tests for CRP are, however, gradually evolving and the sensitivity and suitability for a quantitative assay can improve in the future as new inventions are made [105,106,107,108]. The current commercial kits for CRP based on lateral flow tests exert typically limits of detection around 2.5 mg/L and a working range between 2.5 mg/L and 200 mg/L. The accuracy of the tests depends on a particular device. Typical standard relative errors are around 15% with an inter-assay precision around 20%, but real testing shows the accuracy better: intra accuracy around 4% and inter accuracy slightly above 10% [109]. The overall simplicity (one-step assay with no specific manipulation) with the tested samples and fast procedure finished within 15 min are the major advantages of the commercially available lateral flow tests for CRP. The lateral flow tests are suitable for point-of-care use and use in physician offices or field conditions (field hospitals, refugee camps, etc.). Laboratory use is possible as well, but it is not preferred under these conditions, because the possibility to use it without any analytical devices is a major economic advantage for a workplace or people who are not equipped with laboratory instruments. When calculating costs per assay without taking the price of the instruments into account (for instance, the already equipped laboratories), lateral flow tests are not economically advantageous, because the price per test can be the same or even higher than the average price per reagent and chemical, for instance, for one well of an ELISA kit. The ELISA, CLIA, and other similar methods offer better analytical specifications at the same time. Lateral flow tests for CRP are marginal for standard laboratories due to the mentioned reasons. Nevertheless, they can serve even under standard laboratories for a fast orientation control of other methods or a statim type of test. The final decision on what type of standard tests should be preferred has to be based on considering equipment of the workplace, education of staff, number of tested samples, and time in which the results of the assay should be reached. A survey of basic specifications for the discussed methods is given in Table 2.

The current standard tests and methods for CRP are fully applicable and well accessible for laboratories, and some tests are even for point-of-care conditions. Low sensitivity and high limits of detection are the major drawbacks of the two current methods applicable under point-of-care conditions—immunoturbidimetry and lateral-flow-tests. Considering the facts written in the previous text, the limit of detection for the CRP assay by standard immunoturbidimetry (around 2 mg/L) and lateral flow tests (around 2.5 mg/L) is above the moderate risk of the discussed pathologies represented by a CRP threshold of 1 mg/L. The threshold level can be checked by methods such as ELISA but these methods are not feasible under point-of-care conditions.

The standard point-of-care tests and immunochemical tests are designed for recognition of the common pentameric form of CRP or total CRP not distinguishing monomeric and pentameric forms of CRP. The producers do not provide information about the specificity of the assays for CRP in the monomeric and pentameric forms. Some immunochemical tests for distinguishing the monomeric and pentameric CRP were proposed recently. Zhang and coworkers prepared an ELISA for the quantification of monomeric CRP in biological samples [110]. The assay exerted significant specificity and a limit of detection equal to 1 µg/L.

## 4. New Biosensors and Point-of-Care Bioassays for CRP

Biosensors and bioassays represent an emerging technology that would provide simplicity and suitability for point-of-care conditions typical for the lateral flow tests, but the biosensors and bioassays can also reach analytical specifications such as sensitivity and low limits of detection typical for laboratory methods including ELISA and CLIA. Significant development of these technologies on the CRP assay has taken place in recent years and the area is extensively evolving as new materials and measuring devices are available [111,112,113,114]. The emerging technologies are depicted in various scientific works discussed in the following paragraphs. Evolution of the biosensor and various bioassay devices and bringing them into laboratory praxis could get an analytical device into the hands of patients and physicians that will cover a range of CRP that is not possible by the current technologies under point-of-care conditions. In the present approaches for point-of-care tests manufacturing, there are preferred analytical methods that can be performed in a limited number of steps (ideally, one step accomplishes the assay), are pocket-size or wearable, use a limited number of expensive materials that are reached by the use of miniaturized sensors with a minimal number of used resources for production, and provide analytical specifications that make them competitive to the more elaborative laboratory methods. It is not expected that the biosensors and bioassays will replace the standard laboratory methods. The biosensors and bioassays would serve as a first screen before submitting selected samples to the laboratories. The reliability of the first screen will be the main gain for the CRP-based diagnoses in a healthcare system. In this chapter, standard biosensors (devices combining a biorecognition element with a physicochemical transducer) and novel types of bioassays that are suitable for point-of-care (e.g., assays close to the biosensors where an artificial recognition element such as an aptamer is used) are described.

The recent studies brought results that would be implemented into praxis in the next years. An electrochemical sensor with a molecularly imprinted polymer on screen-printed electrodes was developed by Balayan and coauthors [115]. The sensor was founded on screen-printed electrodes coated with a gold-platinum bimetallic nanomaterial and then a molecularly imprinted polymer prepared from methyl methacrylate and ethylene glycol dimethacrylate with imprinted CRP. The CRP embedded in the final methacrylate membrane was washed out by methanol and acetic acid. The final sensor proved sensitive to CRP in analyzed samples in the presence of a ferrous/ferric mediator when impedance or cyclic voltammetry was applied. The sensor device exerted a limit of detection of 0.1 nmol/L (approximately 120 µg/L) for CRP and a sensitivity of 0.14 µA/nmol/L (approximately 1.2 µA/ng/L). The sensor had quite a good repeatability under various conditions such as pH and temperature and quite a low interference by compounds such as ascorbic acid, cholesterol, glucose, uric acid, or procalcitonin. An electrochemical sensor based on screen-printed electrodes was also developed by Tabrizi and Acedo who chose an RNA aptamer as a recognition element in their assay [116]. The researchers modified the surface of a carbon screen-printed electrode with chitosan containing carbon nanofibers and then with RNA aptamer (dissociation constant for CRP of the aptamer was equal to 0.9 pmol/L) crosslinked by glutaraldehyde and treated with methylene blue. The concentration of CRP was recorded by square wave voltammetry resulting in the linear range of 1–150 pmol/L (approximately 120 ng/L–1.8 µg/L) and a limit of detection of 0.37 pmol/L (approximately 44 ng/L). Screen-printed electrodes were chosen as a platform for biosensor construction where an antibody plays a role as a recognition part (recognition element) of a biosensor device. The biosensor was founded on screen-printed carbon electrodes sprayed and electrodeposited with graphene quantum dots [117]. Immobilization of an antibody specific to CRP followed. The biosensors worked in a label-free mode on the differential pulse voltammetry principle in the presence of electrolyte-containing phosphate-buffered saline and K_3_[Fe(CN)]_6_. A linearity in the range of 0.05–10 µg/L, a limit of quantification of 0.072 µg/L, and a limit of detection of 0.024 µg/L were achieved for the assay. Another electrochemical biosensor for CRP was constructed on the principle of a piezoelectric quartz crystal microbalance sensor with a basic oscillation frequency of 10 MHz [118]. The quartz crystal contained a self-assembled monolayer with an anti-CRP antibody on a gold electrode. The biosensor was suitable for a label-free assay of CRP with a limit of detection of 0.08 mg/L (0.08 µg/mL).

CRP can also be measured by optical biosensor devices allowing the establishment of an analytical method on generally available wearable electronics with the integrated camera. Such devices are currently developed for various biomarkers and their importance will probably grow further [119,120]. An optical biosensor for CRP was developed for instance by Yeh and coworkers [121]. They chose a guided-mode resonance optofluidic biosensing system suitable for point-of-care conditions. Commercial LEDs served as a source of light that was subsequently filtered by a bandpass filter for an outputting range of 500–550 nm. The guided-mode resonance sensor chip was modified by protein A and albumin was used for saturating the surface of the chip to not contain free spaces. An anti-CRP antibody was intercepted by the immobilized protein A. During the assay, CRP was caught by the immobilized anti-CRP antibodies. The anti-CRP antibodies were also applied to the sensor after sample administration to form an immunosandwich. The biosensor exerted a limit of detection of 19.5 µg/L of CRP in an assay cycle lasting 20 min. Another optical biosensor for CRP was developed by Esposito and coworkers [122]. They constructed a fiber optic label-free biosensor where the optical fiber was covered with an anti-CRP antibody and the interaction was recorded as a change of light couple due to a long-period fiber grating. The assay had a limit of detection of 0.15 µg/L and a working range of 1 µg/L–100 mg/L. The interactions can be recorded in real-time, which represents a substantial advantage of the assay. Advanced bioassays can be based not only on an innovative measuring procedure but also on new types of recognition reagents that can provide substantial improvement. Such access was chosen by Al-Enezi and coworkers when preparing an affimer: Eu^3+^ ions chelated by a modified synthetic protein with affinity to the targeted structures such as CRP, carcinoembryonic antigen, and glial fibrillary acidic protein as model biomarkers [123]. The affimer was excited at 395 nm and emitted light at 590 and 615 nm. The fluorescence bioassay detected CRP with a limit of detection of 100 fmol/L (approximately 1.2 pg/L) and it was applicable for a concentration range of 100 fmol/L up to 100 nmol/L (approximately 1.2 pg/L–1.2 µg/L) in an assay lasting 2–3 min. Light reflectance spectroscopy is another platform suitable for CRP assay as reported in the cited papers [124,125]. In a recent application, light reflectance spectroscopy with immobilized anti-CRP antibodies was developed by Tsounidi and coworkers [126]. The assay is composed from two steps. A sample containing CRP was applied in the first step. An anti-CRP antibody was added in the second step and a sandwich complex was formed when CRP was presented in the tested sample. The assay had a dynamic range for CPR equal to 0.05–200 mg/L, a limit of detection of 1 µg/L, a limit of quantification of 2.5 µg/L, and lasted 12 min per assay cycle. Silicon chips are another platform gaining high application potential including the use for CRP assays [127,128,129,130]. A silicon chip for CRP was recently constructed by Psarouli and coworkers [131]. The researchers used an optical method—Mach–Zehnder interferometer and a silicon chip with an immobilized anti-CRP antibody. The interaction of CRP with immobilized antibodies was recorded by a signal represented by a phase shift. The assay exerted a limit of detection of 2.1 µg/L and the achieved signal was linear up to a CRP concentration of 100 µg/L. One assay cycle was finished within 12 min. A survey of the cited biosensors and bioassays for CRP is given in Table 3.

The recently developed biosensors and bioassays discussed in this text represent analytical devices that would be performed in field or point-of-care conditions. Compared to the commercially available point-of-care test such as immunoturbidimetry and lateral flow tests, the new types of biosensors and bioassays exert improved analytical specifications, making them suitable to even recognize CRP levels, indicating a moderate risk of pathologies (typical threshold of 1 mg/L) and even competing with standard laboratory methods such as ELISA. Miniaturization makes the currently developed biosensors and bioassays sized to standards common for wearable electronics. These facts will probably be recognized shortly.

## 5. Conclusions

The relevance of CRP for various diagnoses is a known fact and many commercial tests are manufactured for clinical purposes. There is a growing interest in making the test available for any user and suitable for use under point-of-care conditions. The current analytical tests can provide either high accuracy or sensitivity, but such methods are suitable for use in laboratories only or available for anyone, and sensitivity and other specifications are worsened due to simplicity. The simple tests for point-of-care conditions have substantial flaws such as high limits of detection, the inability to provide accurate information about the concentration of CRP, or the long time needed for one assay. New types of bioassays are developed to replace less reliable or elaborative methods and there are expectations that new generations of devices such as biosensors will provide high sensitivity and good accuracy on the one hand and a user-friendly assay protocol to allow the use of the device under the point-of-care conditions on the other hand. The development and introduction into praxis of the new biosensors and bioassays are also dependent on the availability of new highly sensitive materials such as aptamers or industrially produced antibodies, and sensor platforms allowing the manufacturing of cheap analytical devices giving the results after a one-step assay. When the developed technologies will reach the praxis, patients and the healthcare system would gain a tool that would increase the probability of a proper diagnosis and will reduce costs and negative consequences of inadequate or late therapy.

Further development can also be related to the distinguishing of CRP forms. Because the role of monomeric CRP and a comparison of standard pentameric CRP to the monomeric variant has not been extensively studied in a larger population, it is not fully understood. The next discoveries on monomeric CRP association with various pathogeneses can enhance it as an important biomarker. Further research on analytical methods can take this fact into account and new methods selective to either pentameric or monomeric CRP can be developed.

## Figures and Tables

**Table 1:** Specifications of CRP as a marker.

Feature	Specifications of CRP	References
Structure	protein with five identical subunits each sized 206 amino acids	[16,17,18]
Size	120 kDa (human origin, pentameric structure)	[16]
Occurrence and major source	dominantly produced by hepatocytes and released into the blood	[28,29]
Minor producers	macrophages, lymphocytes, adipocytes, smooth muscle cells, and epithelial cells	[27]
Role in the organism	opsonization of pathogens and other target cells, a part of inflammatory reaction, involved in both classic and alternative complements, and activation of leukocytes	[30,31,32]
Increased level—typical marker	chronic and systemic inflammation, bacterial infection	[40,45,46,49,50,51]
Examples of other relevant reasons for an increased level	heart failures and cardiovascular diseases, osteoarthritis, visceral adiposity, alcohol intake, and some dementias, non-alcoholic fatty liver disease, periodontitis, appendicitis, sepsis, some viral infections such as severe coronavirus disease 2019, candidiasis, and some types of cancer	[35,36,37,38,40,41,42,43,44,47,48,52,53,54,55,56,57,58]
Blood concentrations—typical threshold levels	concentrations under 10 mg/L are taken for normal and levels above 10 mg/L are indicators of pathology	[68]
Other threshold levels and thresholds for hsCRP tests	under 1 mg/L of blood, CRP is a low risk of pathologies such as cardiovascular disorders, 1–3 mg/L is a moderate risk, and a high risk starts at CRP blood levels above 3 mg/L	[70]

**Table 2:** General specification of CRP standard tests.

Assay Principle	User	Quantification	Limit of Detection and Applicability Range	Time Per One Assay	Declared Accuracy
ELISA, CLIA	standard and small laboratories	yes	5 ng/L; 5 ng/L to 50 µg/L	90 min up to one day	relative standard error of 1.5%, inter-assay precision up to 15%
Immunoturbidimetry	Small laboratories, physician offices, point-of-care conditions	yes	2 mg/L; 2 to 150 mg/L	5 to 15 min	relative standard error 4%, inter-assay precision 10%
Lateral flow tests	physician offices, point-of-care conditions, marginal in laboratories	no or semiquantitative	2.5 mg/L; 2.5 to 200 mg/L	15 min	relative standard error of 15%, inter-assay precision around 20%

**Table 3:** Survey of biosensors and bioassays for CRP measurement.

Assay Principle	Recognition Part	Sensor Platform	Limits of detection and Other Reported Specifications	References
Impedimetry, cyclic voltammetry	Molecularly imprinted polymer: CRP imprinted into methacrylate membrane	screen-printed electrodes with metallic nanoparticles	limit of detection 120 µg/L	[115]
Square wave voltammetry	RNA aptamer	screen-printed electrodes with chitosan containing carbon nanofibers and methylene blue	linear range 120 ng/L–1.8 µg/L, limit of detection 44 ng/L	[116]
Differential pulse voltammetry	anti-CRP antibody	screen-printed carbon electrodes sprayed and electrodeposited with graphene quantum dots	linearity 0.05–10 µg/L, limit of quantification 0.072 µg/L, limit of detection 0.024 µg/L	[117]
Piezoelectric	anti-CRP antibody	quartz crystal microbalance	limit of detection 0.08 mg/L	[118]
Optical guided-mode resonance	anti-CRP antibody	guided-mode resonance optofluidic biosensing system	limit of detection 19.5 µg/L, assay time 20 min	[121]
Optical long-period fiber grating	anti-CRP antibody	optical fiber	limit of detection 0.15 µg/L and working range 1 µg/–100 mg/L	[122]
Fluorimetric bioassay	affimer: Eu3+ ions chelated by a modified synthetic protein	fluorimetry	limit of detection 1.2 pg/L, applicable range 1.2 pg/L–1.2 µg/L, time of an assay 2–3 min	[123]
White light reflectance spectroscopy	anti-CRP antibody	light reflectance spectroscopy	dynamic range for 0.05–200 mg/L, limit of detection 1 µg/L	[126]
Mach–Zehnder interferometry	anti-CRP antibody	silicon chip as a part of Mach–Zehnder interferometer	limit of detection 2.1 µg/L, one assay cycle 12 min	[131]

## Data Availability

Not applicable.
